# Supplementary material for: HIVprotI: an integrated web based platform for prediction and design of HIV proteins inhibitors
Source: J Cheminform. 2018 Mar 9;10:12. doi: 10.1186/s13321-018-0266-y (PMC5845081; doi:10.1186/s13321-018-0266-y)
Supplement: Supplementary file 2 — Additional file 2. Source code of HIVProtI web server. [file 13321_2018_266_MOESM2_ESM.zip › HIVprotI_Source-code/hivprotI/weka-3-6-6/doc/weka/gui/visualize/PostscriptWriter.html]

PostscriptWriter


---


|  |  |  |
| --- | --- | --- |
| **Overview** \| **Package** \| **Class** \| **Tree** \| **Deprecated** \| **Index** \| **Help** \| **Weka's home** | |  |
| **PREV CLASS**   **NEXT CLASS** | **FRAMES**    **NO FRAMES**     **All Classes** |  |
| SUMMARY: NESTED \| FIELD \| CONSTR \| METHOD | DETAIL: FIELD \| CONSTR \| METHOD |  |


---


## weka.gui.visualize Class PostscriptWriter

```
java.lang.Object
  weka.gui.visualize.JComponentWriter
      weka.gui.visualize.PostscriptWriter
```

---

``` public class PostscriptWriter extends JComponentWriter ```

This class takes any Component and outputs it to a Postscript file.

**Note:**  
This writer does not work with Components that rely on clipping, like e.g.
scroll lists. Here the complete list is printed, instead of only in the
borders of the scroll list (may overlap other components!). This is due to
the way, clipping is handled in Postscript. There was no easy way around
this issue. :-(

**Version:**
:   $Revision: 7059 $

**Author:**
:   FracPete (fracpete at waikato dot ac dot nz)

**See Also:**: `PostscriptGraphics`

---

**Constructor Summary**

`PostscriptWriter()`
  
          initializes the object

`PostscriptWriter(javax.swing.JComponent c)`
  
          initializes the object with the given Component

`PostscriptWriter(javax.swing.JComponent c,
java.io.File f)`
  
          initializes the object with the given Component and filename


| **Method Summary** | |
| --- | --- |
| `void` | `generateOutput()`             generates the actual output |
| `java.lang.String` | `getDescription()`             returns the name of the writer, to display in the FileChooser. |
| `java.lang.String` | `getExtension()`             returns the extension (incl. |
| `static void` | `main(java.lang.String[] args)`             for testing only |

**Methods inherited from class weka.gui.visualize.JComponentWriter**

`getComponent, getCustomHeight, getCustomWidth, getFile, getScalingEnabled, getUseCustomDimensions, getXScale, getYScale, setComponent, setCustomHeight, setCustomWidth, setFile, setScale, setScalingEnabled, setUseCustomDimensions, toOutput, toOutput, toOutput`

**Methods inherited from class java.lang.Object**

`equals, getClass, hashCode, notify, notifyAll, toString, wait, wait, wait`

**Constructor Detail**

### PostscriptWriter

```
public PostscriptWriter()
```

:   initializes the object

---


### PostscriptWriter

```
public PostscriptWriter(javax.swing.JComponent c)
```

:   initializes the object with the given Component

    **Parameters:**: `c` - the component to print in the output format

---


### PostscriptWriter

```
public PostscriptWriter(javax.swing.JComponent c,
                        java.io.File f)
```

:   initializes the object with the given Component and filename

    **Parameters:**: `c` - the component to print in the output format: `f` - the file to store the output in


**Method Detail**

### getDescription

```
public java.lang.String getDescription()
```

:   returns the name of the writer, to display in the FileChooser.
    must be overridden in the derived class.

    :   **Specified by:**: `getDescription` in class `JComponentWriter`
    :   **Returns:**: the name of the writer

---


### getExtension

```
public java.lang.String getExtension()
```

:   returns the extension (incl. ".") of the output format, to use in the
    FileChooser.
    must be overridden in the derived class.

    :   **Specified by:**: `getExtension` in class `JComponentWriter`
    :   **Returns:**: the file extension

---


### generateOutput

```
public void generateOutput()
                    throws java.lang.Exception
```

:   generates the actual output

    :   **Throws:**: `java.lang.Exception` - if something goes wrong

---


### main

```
public static void main(java.lang.String[] args)
                 throws java.lang.Exception
```

:   for testing only

    :   **Throws:**: `java.lang.Exception`


---


|  |  |  |
| --- | --- | --- |
| **Overview** \| **Package** \| **Class** \| **Tree** \| **Deprecated** \| **Index** \| **Help** \| **Weka's home** | |  |
| **PREV CLASS**   **NEXT CLASS** | **FRAMES**    **NO FRAMES**     **All Classes** |  |
| SUMMARY: NESTED \| FIELD \| CONSTR \| METHOD | DETAIL: FIELD \| CONSTR \| METHOD |  |


---
